# The effect of sacroiliac chiropractic adjustments on innominate angles

**DOI:** 10.4102/hsag.v25i0.1398

**Published:** 2020-11-17

**Authors:** Malany Moodley, Melanie Craig

**Affiliations:** 1 Department of Chiropractic, Faculty of Health Sciences, University of Johannesburg, Johannesburg, South Africa

**Keywords:** sacroiliac, innominate, PALM PALpation Meter, chiropractic, adjustments

## Abstract

**Aim:**

The aim of this study was to determine whether or not a measurable change in the angle of the innominate bone could be identified after a chiropractic sacroiliac adjustment using a ‘PALM PALpation Meter’. Secondly, if a change in the angle of the innominate bone was identified, what was the degree of change in the angle of the innominate bone, induced by the sacroiliac joint (SIJ) adjustment.

**Method:**

This was a true experimental study that consisted of 100 participants who met the inclusion criteria. The participants were randomly allocated to either the treatment or control group. Each group had 50 participants: 25 females and 25 males. Informed consent was obtained from participants prior to commencement of treatment. The treatment group received a chiropractic adjustment based on their specific SIJ dysfunction. The control group was treated with detuned ultrasound therapy (sham treatment).

**Procedure:**

Treatment consisted of a once-off treatment. The angles of the innominate bones were measured bilaterally pre- and post-treatment in both groups. Objective data were collected using the PALM PALpation Meter. Once the dysfunctional SIJ was identified, participants in group 1 were treated with specific chiropractic adjustment techniques based on the restriction. Group 2 participants were treated with detuned ultrasound only.

**Results:**

The results of this study showed that a specific chiropractic adjustment resulted in a measurable change in the angle of the innominate bone (*p* ≤ 0.001). The change in angle was evident bilaterally; however, the side that was adjusted shows the greatest degree of change. The mean change in angle for the treatment group was 2.25° on the side of dysfunction.

**Conclusion:**

The results of this study showed that a specific chiropractic adjustment can have a positive effect on the angles of the innominate bone, resulting in the tilt of the pelvis levelling into what is considered to be its correct anatomical alignment.

## Introduction

Chronic lower back pain (LBP) is the leading cause of disability and loss of productivity on a global level (Crownfield 2013; Ehrlich 2003). The pathophysiology of the world’s most debilitating condition is poorly understood. Research suggests that abnormal biomechanics of the pelvis leads to a functional leg length inequality and myofascial pain syndromes, which ultimately manifest as chronic LBP (Cooperstein & Lew 2009). An anterior or posterior pelvic tilt can change the biomechanics of posture and gait, resulting in chronic LBP because of the development of muscular imbalances. The development of muscle imbalances (in the form of spasm and hypertonicity) is not random, but rather occurs in a logical and predictable pattern (Sweeting 2007).

Lower back pain is considered neither an actual disease nor a diagnostic entity, but rather a collection of symptoms that may present with variable duration and intensity. Worldwide, the incidence and prevalence of LBP has been shown to be similar. This pain is one of the leading causes, if not the number one cause of disability and inability to work (Ehrlich 2003). Lower back pain is considered one of the leading causes of reduced quality of life. It has been noted that acute episodes that last up to 3 months are the most common of presentations; however, recurrent bouts and chronic LBP are more debilitating because of both physical impediment and the negative psychological effects (Ehrlich 2003).

Abnormal biomechanics of the sacroiliac joints (SIJs) may also cause pain and dysfunction syndromes. These syndromes are known to be major contributing factors in the development of LBP. Pain arises because of the poor functioning of the structures (muscles, ligaments and thoracolumbar fascia) that normally stabilise the SIJs (Liebenson 2004).

Sacroiliac joint pain and dysfunction fit into the category of non-specific, benign LBP. It is a syndrome that is diagnosed on physical examination only. The examination is based on pelvic alignment, mobility tests and provocative manoeuvres that stress the SIJ in an attempt to reproduce the patient’s pain. A combination of abnormal pelvic rotation, poor joint locking and imbalances in the muscles and ligaments results in the joint becoming either hypo- or hyper-mobile (Clavel 2011). Joint hypomobility has been attributed to abnormal repetitive stresses which are then maintained by the compressive elastic forces of the ligaments and muscles that normally stabilise the joint (Mitchell et al. 2007).

Sacroiliac joint dysfunction (SIJD) and LBP are often considered two separate entities; however, these two conditions may span a continuum in which SIJD lies at one end and LBP occupies the other end. The conditions may be so interrelated that it is difficult to separate them from each other completely. Lower back pain can be classified as being either acute or chronic. Patients with acute pain are further classified into three subcategories: those with ‘red flag’ conditions involving spinal compression syndromes; those with neurological symptoms and radiculopathies; and those with non-specific or benign LBP. Sacroiliac joint dysfunction fits into the final category (Clavel 2011).

Research has shown that chiropractic treatment can be used to treat and correct functional leg length inequalities that result from abnormal pelvic tilt postures, thereby reducing postural abnormalities, muscular imbalances and LBP (Lawrence et al. 2008). However, there is limited research demonstrating the degree to which a chiropractic adjustment actually does effect a change in the angle of the innominate bones (ilia) of the pelvis. No studies have been done that accurately measure the pre- and post-adjustment angles of the innominate bones.

It has been suggested that chiropractic sacroiliac (SI) adjustments are able to ‘move’ the innominate bones, thereby improving SIJ mobility and functioning (Cooperstein 2010). Despite an extensive search (Pubmed, Medline, ScienceDirect), no literature could be found to substantiate the claim that an adjustment is able to move the ilia of the pelvic ring.

### Incidence and prevalence of sacroiliac joint dysfunction

It is widely accepted that dysfunctional SIJ’s can result in non-specific LBP; however, the prevalence of this disorder has not been well studied. Prevalence studies have been done using either physical examination findings and/or radiological imaging techniques to make a definitive diagnosis (Cohen 2005). A retrospective study by Bernard and Kirkaldy-Willis (1987) found a 22.5% prevalence rate in 1293 adult patients presenting with non-specific LBP. During this study the diagnosis of SIJD was based on physical examination and clinical findings (Bernard & Kirkaldy-Willis 1987).

Schwarzer, Aprill and Bogduk (1995), conducted a prevalence study involving 43 patients that presented with LBP. They used fluoroscopically guided SIJ injections. In the study the research team used three criteria to diagnose SIJ pain: analgesic response to local anaesthetic (LA), abnormalities on computed tomography (CT) scanning and pain reproduction during joint distension. The study showed that 30% of the participants experienced pain because of SIJ involvement (Schwarzer et al. 1995).

In a prevalence study done by Maigne, Aivaliklis and Pfefer (1996), 54 patients presented with unilateral LBP. Lidocaine blocks were injected into the SIJ. Nineteen patients had a positive response, with a greater than 75% reduction in pain, to the lidocaine screening block. These patients were considered to have true SIJ pain (Maigne et al. 1996).

Based on these studies, the prevalence of SIJ pain in carefully screened LBP patients appears to be in the 15% – 25% range (Cohen 2005).

### Understanding sacroiliac joint dysfunction

The literature suggests that 85% of LBP is classified as non-specific LBP. Sacroiliac joint dysfunction is considered to be a major contribution factor in non-specific LBP (Clavel 2011). It has been estimated that 22.5% of all non-specific LBP is because of SIJD (Joseph et al. 2014). There are a multitude of factors that can contribute to the onset and recovery of non-specific LBP. It has been noted that there is often a direct relationship between pain, risk factors and chronicity of LBP (Clavel 2011).

According to Joseph et al. (2014), SJID refers to any altered or impaired biomechanical functioning of the SIJ, and the consequential changes that are noted in the muscles and ligaments surrounding the joint. It is a musculoskeletal disorder where the joint is biomechanically incompetent to transmit load normally in the absence of a demonstrable pathology (Joseph et al. 2014).

Sacroiliac joint dysfunction is vastly different from SIJ pathology. The two may or may not be linked or associated with each other, but are not exclusive to each other. There are a great number of pathological conditions that can affect the SIJ. Each of these conditions is well documented and described in the literature. Most pathological processes that affect the SIJ can be positivity diagnosed with the use of radiographic imaging, blood tests and clinical findings (Clavel 2011).

When contrasting SIJD and SIJ pain syndromes, diagnosis is based on patient history, clinical examination, pelvic alignment and mobility, and provocative tests that stress the SIJ. There are currently no objective diagnostic tests that can be used to diagnose SIJD. Dysfunction may or may not present with pain. Sacroiliac joint dysfunction results from a combination of altered or abnormal biomechanics, joint locking and hypomobility. There may also be associated muscle imbalances (Clavel 2011).

There are no muscles that act directly on the SIJ, but this joint is placed under indirect strain by the muscles that act on the pelvic girdle. These muscles hold the pelvis in a rotated position to stabilise the lumbo-sacral spine. The most common pattern of stabilisation is right anterior and left posterior rotation of the innominate bones. This rotation will result in sacral torsion leading to compensation in the lumbar spine (Clavel 2011).

If there is habitual activation and shortening of the right anterior hip girdle muscles, there will be maintained rotation of the innominates and sacral torsion. The same can be seen in an acute guarding response because of trauma. This can lead to weakness and/or deconditioning of the core stabiliser muscles (erector spinae, quadratus lumborum, external and internal oblique muscles) resulting in this becoming a dominate pattern (Clavel 2011).

When this pattern ensues for an extended period of time, it can result in shortening and/or spasm of the right piriformis muscle. This will cause the sacrum to be pulled against the ilium essentially locking the joint and decreasing joint movement (Clavel 2011).

### The PALM PALpation Meter

The PALM PALpation Meter (PPM) allows an examiner to test for skeletal asymmetry using palpation augmented by the objectivity and reliability of inclinometer and calliper measurements. The calliper determines the distance in centimetres between the fingers and the inclinometer determines the inclination in degrees between them. Using the unique slide rule calculator, the examiner can discover the height discrepancy between the two chosen landmarks.

Petrone et al. (2003) conducted a research study to measure the accuracy of the PPM in measuring pelvic crest heights. The study concluded that the PPM was a reliable and valid instrument for measuring pelvic crest height differences. The study compared measurements obtained with the instrument to those obtained from standard radiographic films and 98% accuracy was achieved (Petrone et al. 2003).

In two other studies cited by Herrington (2011), the inter-rater reliability (*r*) and standard error of measurement (SEM) were assessed. In both studies *r* = 0.98–0.99 and SEM = 0.44º – 0.47º. Both studies concluded that the device was valid and reliable at measuring pelvic inclination (Herrington 2011).

In the Herrington study (2011), participants were positioned with their feet 30 cm apart, standing on a flat, level surface looking directly at a point ahead of them to prevent postural sway. Their body weight was evenly distributed on both feet and their arms folded across their chest. The examiner located the anterior superior iliac spine (ASIS) and posterior superior iliac spine (PSIS) via static bony palpation and marked them with a skin marker. Once the appropriate anatomical landmarks were located and marked, the calliper arms of the PPM were aligned to these marks and the angle of inclination was measured (Herrington 2011).

## Materials and methods

### Selection criteria

The design of the study was a true experimental (pre-test, post-test only design) as there is limited research regarding the angle of the innominate bones before and after a chiropractic adjustment. Convenience sampling was used. As there was little pre-existing research for sample size calculations, recommendations on sample sizes for pilot studies were used. Planning for a future study with 80% power, 50 participants in each arm, is suggested for small effect sizes (Whitehead et al. 2016).

The research sample consisted of 100 participants (*N* = 100) between the ages of 18 and 45. Participants were eligible to be part of the study if they meet the inclusion and were excluded from the study if they presented with any of the exclusion criteria. The participants were separated into two groups. Participants were randomly allocated to a group to ensuring gender equality ratios. Each group had 50 participants (*n* = 50): 25 male and 25 female. The treatment group received a chiropractic adjustment based on their specific SIJD. The control group was treated with detuned ultrasound therapy (sham treatment).

### Methodology

Members of the public and students who frequent the University of Johannesburg (UJ) Chiropractic clinic were invited to participate in the study. Recruitment was done through word of mouth and by advertisements that were strategically placed around the UJ Doornfontein Campus and Chiropractic Day Clinic. All participants accepted into the study needed to meet the inclusion and exclusion criteria. The study took place at the UJ Chiropractic Day Clinic. Each participant received a once-off treatment. There was no follow-up consultation related to the study.

#### Inclusion criteria

- male or female
- aged 18–45 years; prior to the onset of age-related degenerative changes
- history non-specific LBP and/or SIJ dysfunction
- presenting with SIJ hypomobility and dysfunction; determined using Gillett’s test, the standing flexion test, seated flexion test and the leg length test.

#### Exclusion criteria

- were pregnant or had recently given birth because joint laxity increases during pregnancy. This increase may still be apparent for up to 6 weeks postpartum
- had hip joint or spinal pathologies that are contra-indicated for spinal manipulative
- have undergone spinal fusion surgery of the lumbar spine or sacrum
- Have undergone hip replacement surgery.

#### Procedure

All participants were assessed in a neutral standing position on a level surface. Each participant was measured on the same square metre of floor space. The levelness of the floor was assessed using a carpenter’s spirit level. There were foot placement markings placed into the square metre of floor so that all participants stood in the same place and position for both the pre- and post-treatment measurements.

The PPM combines the features of a calliper and an inclinometer into one instrument. The ASIS and PSIS were located bilaterally through manual static bony palpation, and marked with a permanent marker. The calliper arms of the PPM were placed on the homo-lateral (situated on the same side of the body) ASIS and PSIS. The inclinometer measured the tilt angle of the innominate bones in degrees. All participants had their innominate bone tilt angles measured bilaterally. The measurements were recorded on the data sheet. The angles were measured three times and averaged for accuracy (e.g. 3° + 2° + 4° = 9°/3 = 3°).

The participants’ SIJs were motion palpated using Gillett’s test, the standing flexion test, seated flexion test and the prone leg length test to determine the side of dysfunction (Bergman & Peterson 2011). All four of the above palpation methods were used to accurately determine the side of dysfunction (the restricted side [RS]). Once the dysfunctional or hypomobile joint was located that dysfunctional joint was treated.

Participants in group 1 were adjusted using the side posture chiropractic adjustment that was specifically indicated for their specific SIJ restriction/dysfunction. The participant was placed in a lateral recumbent position with the lesion side up. The researcher took contact on the restricted segment and adjusted the joint using a low amplitude, high velocity thrust. A diversified thigh-ilio-deltoid chiropractic adjustment was performed to improve joint functioning (Schafer & Faye 1998).

Participants in group 2 were placed in a prone position with the SIJs exposed. Conductive gel was applied over the area of concern, that is, the dysfunctional SIJ. The SIJs were motion palpated using the same method as for the treatment group to determine the dysfunctional joint. The participant was placed in a position so that they were unable to see the ultrasound unit settings. The timer was set for 3 min, but the current remained off to ensure zero therapeutic outcomes. The ultrasound head was applied over the SIJ in a circular motion simulating a normal treatment. Once the study was concluded, participants who received the detuned ultrasound were afforded the opportunity to decide if they would like to receive the chiropractic adjustment treatment.

After the treatment, the angles of the innominate bones were re-measured using the PPM in the same method that was used for the pre-treatment measurements. The same ASIS and PSIS points that were marked pre-treatment with the permanent marker were used to ensure accuracy of the measurements. The participant stood on the same square metre of floor that was used for the pre-treatment measurements. The innominate angles were again measured three times and averaged for accuracy. The post-treatment measurements were recorded in the data sheet.

The objective data for this study were measured using the PPM before and after the chiropractic adjustment. Measurements were in degrees. Pre- and post-treatment measurements were compared for any changes. All objective measurements were obtained using the methodology utilised and validated by Herrington (2011). The PPM is a valid and reliable tool used to measure the innominate angles (Herrington 2011).

### Ethical consideration

Ethical approval of the study was granted by Faculty of Health Sciences Ethics Committee University of Johannesburg, Ethical Clearance Number: AEC01-22-2014, 20/03/2014

## Results

The statistical analysis was conducted with a 95% confidence level. The test for normality was done using the Shapiro–Wilk tests, with a statistical significant level set with a *p*-value of less than 0.05 (*p* < 0.05). Because of the small sample size of each group being compared in this study, no assumption can be made with respect to the population as a whole. To establish statistical significance, the probability level (*p*-value) was set at 0.05. From the *p*-values of this test, we can reject the alternative hypothesis and conclude that the data come from a normal distribution.

### Intra-group analysis

The intra-group analysis was done using the parametric ANOVA mixed between–within test and the non-parametric Wilcoxon-signed rank tests. Both types of analysis were performed to show agreement of results, but only parametric results are discussed.

The ANOVA mixed between–within subject’s analysis of variance was conducted to assess the impact of adjustments versus detuned ultrasound on participant’s innominate bone angles pre- and post-treatment. The test showed that there was a statistically significant difference between group 1 and group 2 post-treatment (*p* ≤ 0.001).

A Wilcoxon-signed rank test revealed a statistically significant change in the angle of the innominate bone in group 1 (*p* ≤ 0.001). There was no statistically significant change in the angles of the innominate bones in group 2 (*p* = 0.418*).*

#### Group 1

**FIGURE 1:**
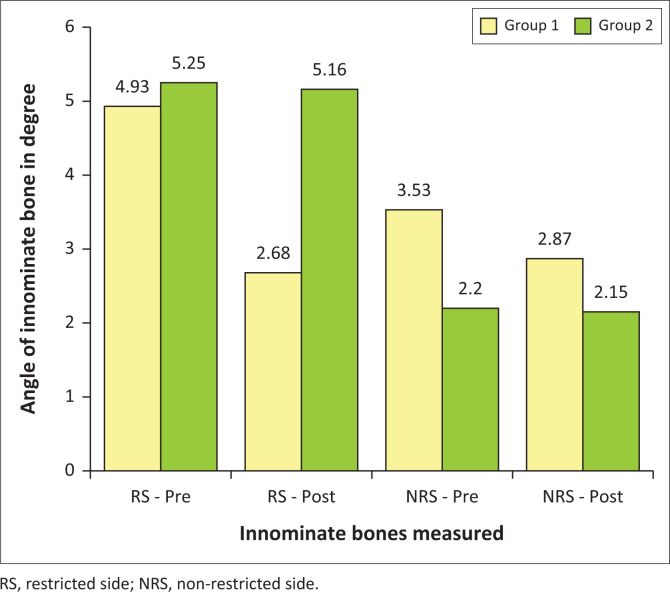
PALM PALpation Meter readings comparing mean values in degrees.

The bar graph (Figure 1) shows that the mean value for group 1 on the RS pre-treatment was 4.93° and post-treatment was 2.68°. There was a mean change of 2.25° on the RS. The non-restricted side (NRS) pre-treatment mean was 3.53° and post-treatment mean was 2.87°, showing a change of 0.66° on the NRS. The angle of the innominate bone showed a mean change of 45.6% on the RS and the 18.7% on the NRS between pre- and post-treatment readings. The change in angle was deemed statistically significant on the RS (*p* ≤ 0.001). All the above-mentioned intragroup analysis can be seen in Table 1.

**TABLE 1:** Intragroup analysis for *p*-value, pre- and post-treatment means, mean difference and percentage change for group 1.

Group 1	*p*	Pre-treatment mean	Post-treatment mean	Mean difference	Percentage change
RS change	0.000	4.93°	2.68°	2.25°	45.6
NRS change	0.000	3.53°	2.87°	0.66°	18.7

RS, restricted side; NRS, non-restricted side.

#### Group 2

As noted in Figure 1, the angles of the innominate bones in group 2 had a RS mean value of 5.25° pre-treatment and 5.16° post-treatment. The angle of the innominate bones on the NRS mean value pre-treatment mean was 2.20° and post-treatment mean was 2.15°. The change in angle on the RS was 0.09° and on the NRS was 0.05°. This indicates that there was a mean change of 1.65% on the RS and 2.12% on the NRS in the average angles of the innominate bones in the control group. The change in angle was deemed not statistically significant on the RS (*p* = 0.418). All of the above-mentioned intragroup analyses can be seen in Table 2.

**TABLE 2:** Intragroup analysis for *p*-value, pre- and post-treatment means, mean difference and percentage change for group 2.

Group 2	*p*	Pre-treatment mean	Post-treatment mean	Mean difference	Percentage change
RS change	0.418	5.25°	5.16°	0.09°	1.65
NRS change	0.418	2.20°	2.15°	0.05°	2.12

RS, restricted side; NRS, non-restricted side.

### Inter-group analysis

The non-parametric Mann–Whitney *U* test was used to compare the sampled data from group 1 and group 2. The test revealed a statistically significant difference between the groups for the post-treatment measurement (*p* ≤ 0.001).

**FIGURE 2:**
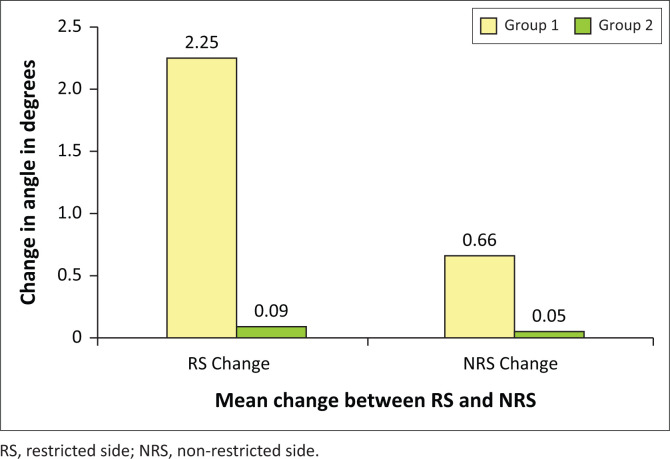
Mean value changes in angles of the innominate bones measured in degrees.

The bar graph shows that the mean change for group 1 on the RS was 2.25° and on the NRS was 0.66°. For group 2, the mean change in angle was 0.09° on the RS and 0.05° on the NRS. Figure 2 illustrates a bar graph comparing the mean change in the angles of the innominate bones between the two groups. The yellow bars represent the mean changes for group 1 and the green bars represent the mean changes for group 2. The *x*-axis shows the mean angles for the RS and NRS, and the *y*-axis shows the mean change in angle of the innominate bone in degrees.

## Discussion

When considering the data that were obtained from this study, it is important to understand how the biomechanics of the adjustment technique can influence and ultimately change the angle of the innominate bone. A sacroiliac dysfunction is identified through palpation for joint play, whilst the patient and participant is both standing and seated. Palpation will give the practitioner a general idea of where in the joint the fixation is located. The SIJs may be fixated in either flexion or extension. The goal of the adjustment is to restore normal functioning of the joint. Once the joint has been evaluated and the type of fixation determined the joint can be treated with a chiropractic adjustment specifically for that restriction (Gatterman 1990). During this study only the thigh-ilio-deltoid adjustment was utilised.

The SIJs are diarthrodial joints that exhibit little movement and are mainly involved in weight bearing. It is, however, imperative that normal functioning is maintained no matter how small the joints range of motion is. The joints can be subjected to reversible fixations within their limited range of motion. These fixations are frequently at the extreme ends of the range of movement (Gatterman 1990).

One of the defining features of the diversified technique is the ability to localise adjustments via the use of pre-adjustment joint tension. Adjustment forces are localised to a specific joint, resulting in an illumination of the joint dysfunction (Esposito & Philipson 2005).

Movement of the SIJ is primarily in the sagittal plane along the angle of the joint surfaces. In treating SI dysfunction, contact is made on either the ilium or the sacrum. The adjustments may be applied in a variety of patient and participant positions, and incorporate methods that establish contact on both sides of the articular surfaces (Byfield 2012). During this study only an ilium contact was used with the patient and participant in a side posture; however, both are discussed for completeness of information.

According to Byfield (2012), the thrust force of a sacroiliac adjustment has been recorded in the range of 200 N – 550 N, which roughly equates to one-third to three-quarters of the average man’s body weight. The ability of a practitioner to achieve these forces has little to do with the height and weight of the practitioner, but more to do with the strength of the adjuster or doctor and speed of the adjustment (Bergman & Peterson 2011).

Asymmetrical tension across the SIJ is the main mechanism of dysfunction pertaining to this region. Forces that are applied to the pelvic bones during the adjustment are dissipated to the soft tissues that surround the joints before they actually reach the joint surfaces themselves. Absolute joint isolation during SI adjustments is difficult because of the complex arrangement of supporting soft tissue, and therefore adjustments that are delivered to the SIJs may have an effect on the lumbosacral articulations and vice versa (Bergman & Peterson 2011).

Several different adjustment techniques have been described by both chiropractors and physiotherapists over the years. To achieve the best results for the patient and participant, the practitioner must be proficient in a number a different mobilisation and adjustment skills, which can be adjusted depending on the clinical presentation of the patient and participant. Most techniques are a variation on a common theme based on the movements of nutation and counter nutation. The reciprocal movements of the innominate bones in relation to the sacrum. The neuro-biomechanical model of the SIJs and the surrounding structures take into consideration the mechanical influences of all the stabilising soft tissue structures that influence the biomechanical movement and kinematic chains that the joints are a part of (Esposito & Philipson 2005).

When one considers the SIJs and the dysfunction associated with them, the biomechanics of the joints must be considered for both diagnosis and treatment purposes. The mechanical influence of musculo-ligamentous interplay is closely associated with various kinematic chains that influence the SIJ, and the irritation of these surrounding soft tissue structures can influence mechanical irritation and ultimately the functioning of the joint. It is therefore important for a practitioner not only to address the joint fixations through adjustment techniques, but also to treat the surrounding soft tissue structure that stabilises this joint via mobilisation and modalities. Some of the most common disorders of the pelvic ring are myofascial pain syndromes and postural asymmetries (Byfield 2012).

Sacroiliac joint dysfunction is characterised as restricted motion of the ilium on the sacrum. The joint dysfunction may be as a result of muscular imbalances, leg length inequalities or acute trauma. The SIJD can manifest as LBP, SIJ pain or pelvic torsion (Bergman & Petersen 2011).

When the dysfunction results in pelvic torsion, it affects the angles of the innominate bones. This can result in abnormal biomechanics of the hip, SIJs and lumbar spine. This altered biomechanics will result in perpetuation of pain syndromes (Lee & Yoo 2012).

When one has an understanding of the biomechanics of the adjustment, how and where the practitioner contacts the patient and participant, the results are explained. The results of this study have shown that a specific chiropractic adjustment does result in a measurable change in the angle of the innominate bone. The change in angle is evident bilaterally; however, the side that was adjusted shows the greatest degree of change.

## Conclusion

The results of this study suggest that joint dysfunction can possibly be determined using the PPM to determine the side of the dysfunctional SIJ as well as the degree to which the innominate bone is tilted either anteriorly or posteriorly.

The aim of this study was to determine whether or not a specific chiropractic adjustment can cause a change in the angle of the innominate bone. The effectiveness of the adjustment was determined by using the objective measurements obtained from the PPM.

On analysis of the data collected it was determined that there was a statistically significant change in the objective data that were collected from group 1 being the treatment group. The data collected from group 2, the control group, showed no statistical significance. The conclusion that is drawn from this analysis is that a specific chiropractic adjustment can result in a change in the angle of the innominate bone.

When considering the structure of the pelvic girdle, it is imperative to remember that the sacrum and innominates are biomechanically linked in a closed chain system. Therefore, this system should be considered as one mechanical unit. If a force, that produces a change, is exerted on any one specific area of the system, a resultant force will be expressed in other areas (Hertling & Kessler 2006).

Once the data from the study were analysed, it showed that the angle of the innominate bones in group 1 changed on both the RS and the NRS. The largest change was noted on the RS, but both angles showed a statistically significant change. From this we can deduce that even if only the dysfunctional joint is adjusted, a change is effected on the normal side as well.

The results of this study show that a specific chiropractic adjustment can have a positive effect on the angles of the innominate bone. Resulting in the tilt of the pelvis normalising into what is considered to be its correct anatomical alignment.

A secondary outcome of this study was to determine the degree to which an adjustment can change the angle of the innominate bone. The study showed that in a joint that has limited range of motion, estimated at only 6°, a mean change of 2.89° (48.2%) is statistically significant (Miller et al. 2013; Vleeming et al. 2012).

Research has showed that manual palpation of the SIJD is subjective, and inter-examiner reliability studies have shown poor results (Fryer, McPherson & O’Keefe 2005). The analysis of the data obtained in this study suggests that the use of the PPM may be suggested as an objective measure of assessing SIJD. This study has illustrated that the dysfunctional side can be determined by the larger innominate angle when measured with the PPM.

## Recommendations

The following recommendations can be used to further improve the results that were obtained during this study as well as suggestions for continuations of the study and possible similar studies:

### Sample size

A larger sample size for both groups could be instituted; this would provide for better statistical analysis on the collected data. If the samples sizes were larger, it would provide a greater degree of validity to the study.

### Follow-up consultations

It is recommended that when doing a similar study, follow-up consultations are instituted. This would be to ascertain if the change in angle induced by the adjustment is maintained or if the innominate bones revert back to their dysfunctional state. Further research can also be conducted on the physiological and neurophysiological impact on the tissues surrounding the SIJs pre- and post-adjustment.

### Consideration of posture and leg length inequality

This study did not take into consideration how abnormal posture, such as scoliosis, or anatomical and functional leg length inequalities effect the angle of the innominate bone. It is recommended that in future studies the angle of the innominate bone be correlated with the degree of postural abnormality or leg length inequality.

### Consideration of muscular imbalances

The results of muscular imbalances of muscles that have an effect on the SIJ were not considered in this study. It is recommended that in future studies the muscle tone and strength of the muscles that affect the SIJ be taken into consideration.

### Effects of range of motion

The study could be performed to investigate to what extent the change in the angle of the innominate bone effects that range of motion of the joints above and below it. How does the change in angle of the innominate bone effect hip joint and lumbar spine range of motion.

### Effects on lower back pain

A study could be performed to investigate to what degree the change in angle of the innominate bone as an effect on the perceived pain that is experience in patients with non-specific LBP.
